# Progress and Challenges in Quantifying Carbonyl-Metabolomic Phenomes with LC-MS/MS

**DOI:** 10.3390/molecules26206147

**Published:** 2021-10-12

**Authors:** Yuting Sun, Huiru Tang, Yulan Wang

**Affiliations:** 1 Key Laboratory of Magnetic Resonance in Biological Systems, State Key Laboratory of Magnetic Resonance and Atomic and Molecular Physics, National Centre for Magnetic Resonance in Wuhan, Wuhan Institute of Physics and Mathematics, Innovation Academy for Precision Measurement Science and Technology, Chinese Academy of Sciences, Wuhan 430071, China; sunyuting18@mails.ucas.ac.cn; 2 University of Chinese Academy of Sciences, Beijing 10049, China; 3 State Key Laboratory of Genetic Engineering, Zhongshan Hospital and School of Life Sciences, Human Phenome Institute, Metabonomics and Systems Biology Laboratory at Shanghai International Centre for Molecular Phenomics, Fudan University, Shanghai 200438, China; Huiru_tang@fudan.edu.cn; 4 Singapore Phenome Centre, Lee Kong Chian School of Medicine, Nanyang Technological University, Singapore 639798, Singapore

**Keywords:** carbonyl-containing metabolites, RPLC-ESI-MS, stable isotope-coded derivatization, absolute quantification, submetabolome profiling

## Abstract

Carbonyl-containing metabolites widely exist in biological samples and have important physiological functions. Thus, accurate and sensitive quantitative analysis of carbonyl-containing metabolites is crucial to provide insight into metabolic pathways as well as disease mechanisms. Although reversed phase liquid chromatography electrospray ionization mass spectrometry (RPLC-ESI-MS) is widely used due to the powerful separation capability of RPLC and high specificity and sensitivity of MS, but it is often challenging to directly analyze carbonyl-containing metabolites using RPLC-ESI-MS due to the poor ionization efficiency of neutral carbonyl groups in ESI. Modification of carbonyl-containing metabolites by a chemical derivatization strategy can overcome the obstacle of sensitivity; however, it is insufficient to achieve accurate quantification due to instrument drift and matrix effects. The emergence of stable isotope-coded derivatization (ICD) provides a good solution to the problems encountered above. Thus, LC-MS methods that utilize ICD have been applied in metabolomics including quantitative targeted analysis and untargeted profiling analysis. In addition, ICD makes multiplex or multichannel submetabolome analysis possible, which not only reduces instrument running time but also avoids the variation of MS response. In this review, representative derivatization reagents and typical applications in absolute quantification and submetabolome profiling are discussed to highlight the superiority of the ICD strategy for detection of carbonyl-containing metabolites.

## 1. Introduction

Carbonyl-containing metabolites are characterized by the presence of an acyl group (R-C=O), such as aldehydes, ketones, keto acids, ketosteroids, and saccharides. Carbonyl-containing metabolites widely exist in biological samples and participate in many metabolic pathways, such as glycolysis, TCA cycle, fatty acid β-oxidation, and hormone synthesis. These metabolites have important physiological functions. For example, as products of lipid peroxidation induced by oxidative stress, several endogenous aldehydes and ketones, such as acrolein, 4-hydroxy-2-nonenal (HNE), and malondialdehyde (MDA), can exacerbate oxidative damage [1], resulting in changes in cell structure and dysfunction [2] or triggering various inflammatory and immune responses [3,4]. In addition, ketosteroids are a class of steroid hormones generated by the metabolism of cholesterol in the adrenal cortex, gonads, and placenta [5]. The metabolic disruptions of ketosteroids are associated with various endocrine diseases [6] and cancer [7], for example, the excessive secretion of aldosterone caused by primary aldosteronism (PA) can lead to elevated blood pressure and adversely affect the cardiovascular system [8]. In addition, saccharides occupy an important position in central carbon metabolism, including glycolysis and pentose phosphate pathways, which maintain the most basic life activities in living organisms [9]. Abnormal glucose metabolism behavior allows tumor cells to escape the normal process of apoptosis and enhance their proliferation and migration capabilities, which is called the Warburg effect and is a key factor in the pathogenesis of tumors [10]. Therefore, carbonyl-containing metabolites are seen as potential biomarkers [11] of many diseases such as diabetes [12], cardiovascular diseases [13], neurodegenerative diseases [14], and lung cancer [15]. Thus, accurate and sensitive quantification of carbonyl-containing metabolites is crucial to elucidate their biological function in these diseases.

Although studies on changes in the concentration of carbonyl-containing metabolites in biological fluids and tissues can provide insight into metabolic pathways as well as disease mechanisms, quantitative analysis of them remains a challenge. Firstly, there are thousands of carbonyl-containing metabolites in biological fluids and tissues which often exist in combination with other functional groups. Due to their different chemical structures, there is high diversity of physicochemical properties, such as solubility, volatility, and stability. Secondly, the concentrations of carbonyl-containing metabolites are low in some biological matrixes, for example, the concentration of pyruvaldehyde in the blood of healthy adults is 0.65 μM [16], while the concentration of androsterone is only 0.00065 μM [17], which is only one-thousandth of that of pyruvaldehyde. Quantifying these relatively low abundance endogenous metabolites from biological fluids is challenging due to stringent requirements for sensitivity and specificity. Thirdly, the diverse range of carbonyl-containing metabolites and low concentrations are further complicated by the high dynamic range in concentration of metabolites by physiological status. For example, the levels of testosterone are typically related to gender and developmental stage and physiological concentrations of testosterone in the serum of adult women are only 5–10% of those in adult men and are much lower in infants and children of both sexes [18]. Finally, due to the inherent variations of sample matrix such as salt content in biological samples (e.g., urine and sweat), measurement of carbonyl-containing metabolites is further compromised by matrix effects, leading to reduced accuracy of quantification [19].

Various methods have been developed to measure carbonyl-containing metabolites, including immunoassay, nuclear magnetic resonance (NMR), and mass spectrometry (MS). Immunoassay [20] is the most used method for steroid hormone analysis, which is relatively simple and easy to perform. However, it lacks sensitivity and specificity, especially when quantifying different steroid metabolites with similar chemical structures and all at low concentrations [21]. Due to cross-reactivity and matrix interference, the inter-assay variability of immunoassay limits quantitative accuracy [22]. Furthermore, an immunoassay is based on a specific antigen–antibody reaction, making it difficult to achieve high-throughput simultaneous analysis of multiple metabolites [23].

NMR is a preferred method for the detection of a broad spectrum of metabolites. In a typical biological sample, almost all metabolites will produce ^1^H NMR peaks, if their concentrations are above the detection limit. The advantage of NMR is that biological fluids do not require any physical or chemical treatment before analysis. The non-destructive nature of NMR also makes it possible to observe the dynamics and sequestration of metabolites in tissue samples [24]. However, the ^1^H NMR spectrum of a biological sample is an overlay of the spectra of all metabolites in the sample, which may lead to inaccurate concentration determination. In addition, the ability to detect low concentrations of metabolites is limited as compared with an MS-based analysis [25]. The limit of detection of NMR is usually in the millimolar to micromolar range, making NMR unsuitable for some carbonyl-containing metabolites, such as ketosteroids with concentrations below the nanomolar level.

MS is a detection method with high specificity and sensitivity, which is usually combined with a high-resolution separation technique, such as gas chromatography or liquid chromatography. Due to differences in separation and ionization mechanisms, both separation techniques have their own pros and cons. GC-MS is highly sensitive and specific for separation and detection of volatile aldehyde and ketones, such as, formaldehyde and acetone. In addition, high reproducibility fragmentation pattern and retention time of metabolites and low instrumental-based variability in GC-MS allow for matching with an established metabolome database for metabolite identification [26]. However, derivatization is required for nonvolatile metabolites, which is tedious and labor intensive [27]. The majority of carbonyl-containing metabolites are polar, nonvolatile, and require derivatization prior to GC analysis; certain carbonyl-containing metabolites, such as keto acids, are inherently unstable to heat and light and are easily decarboxylated [28], making it difficult to analyze by GC-MS.

LC-MS has become the most versatile analytical approach for metabolomics, including untargeted metabolic profiling [29] and targeted metabolite quantification [30], as it permits the detection of a broad range of different chemical properties of metabolites in varying concentrations. The type of chromatography column and mobile phase are the key factors for LC separation for metabolomics. Reverse-phase (RP) columns have the advantages of high resolution, good repeatability, and extensive metabolite coverage, in particular, for the analysis of low-polarity metabolites [31]. Moreover, the mobile phase gradient for RPLC usually starts with a high percentage of water and less organic solvent, which makes it, currently, by far most commonly used in metabolomics as it is compatible with the analysis of aqueous biological samples [32]. The selection of the ionization mode is important because it determines the types of metabolites being detected. Electrospray ionization (ESI) is the preferred ionization mode, because it requires lower temperatures for ionization than other ionization modes, and therefore it can be utilized for thermally unstable metabolites [33].

Although RPLC-ESI-MS integrates the excellent separation capability of RPLC, good versatility of ESI and high specificity and sensitivity of MS detection, it is often challenging to directly analyze carbonyl-containing metabolites due to poor ionization efficiency of neutral carbonyl groups by ESI. In addition, the high polarity of some carbonyl-containing metabolites exhibits poor chromatographic retention in RPLC. In this regard, chemical derivatization prior to LC-MS detection is an emerging strategy for overcoming the obstacles to analyzing carbonyl-containing metabolites [34]. Chemical derivatization-based LC-MS has been developed since the 1980s [35] and the introduction of chemical moiety could convert the carbonyl-containing metabolites into MS-detectable and LC-separable derivatives. For example, Zhao et al. [36] used dansylhydrazine (Dns-Hz) as a derivatizing reagent for labeling of carbonyl-containing metabolites, so that acetaldehyde can be ionized after derivatization and its MS signals significantly increased. In addition, chemical derivatization facilitates chromatographic separation of isomeric metabolites, such as glucose 6-phosphate and fructose 6-phosphate [37].

There are two advantages of chemical derivatization in mass spectrometry quantification. First, chemical derivatization can enhance the MS response and LC separation of metabolite species that have low abundance, poor chromatographic behavior, or are difficult to ionize. Second, chemical isotope labeling could solve the problem of concentration-independent MS signal differences caused by ionization efficiency. This is due to instrument drift and matrix effects produced by the co-eluting components, which cause fluctuations in the ionization efficiency of metabolites in ESI, thereby, resulting in a variable response in LC-ESI-MS analysis [38]. In general, an internal standard (IS) is needed to ensure the accuracy and precision of quantification because of its identical physicochemical properties and similar chromatographic behavior as the metabolites. However, it is impractical to provide the stable isotope-labeled analogs for all target metabolites due to limited commercial availability, labor-intensive syntheses, and high cost [39]. Thus, a more reliable strategy is urgently required. The emergence of ICD can solve the problems outlined above. In this strategy, the stable isotope-coded moiety of derivatization reagent is used to label standard metabolites to form derivatives, which acts as ISs. This approach can generate isotope ISs for all the target metabolites simultaneously in the derivatization step.

Several excellent reviews have focused on isotope-coded derivatization strategies and their applications in global metabolomics or targeted small-molecular weight compounds [39,40,41,42,43]. In this review, we focus on the detection of carbonyl-containing metabolites and present the general principles and properties of representative derivatization reagents, as well as their advantages and shortcomings. In addition, typical applications in absolute quantification and submetabolome profiling are discussed to highlight the superiority of the ICD strategy for detection of carbonyl-containing metabolites.

## 2. Types of Derivatization Reagents for Carbonyl-Containing Metabolites

**Figure 1:**
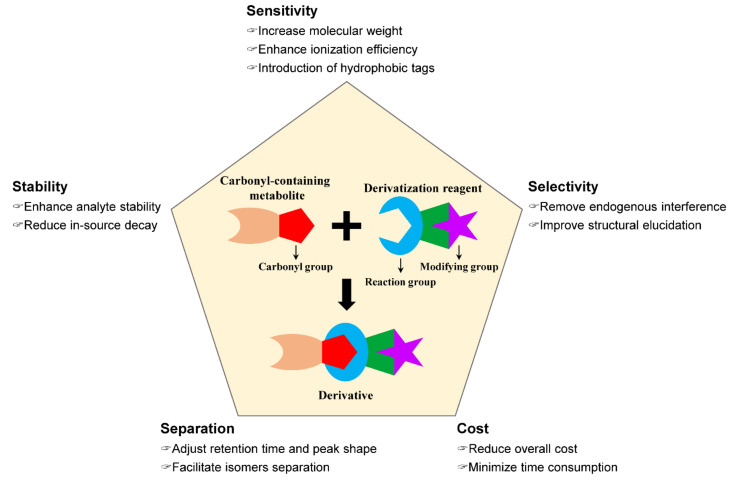
Key factors of an excellent chemical derivatization reagent.

As described above, modification of carbonyl-containing metabolites by a chemical derivatization strategy can convert them into LC-separable and MS-detectable derivatives. As shown in Figure 1, an excellent chemical derivatization reagent should be able to (1) enhance the stability of analytes by modifying the unstable moiety of analytes and reduce its in-source decay during MS detection [44,45], (2) improve the detection selectivity through removing endogenous interference or aiding structural elucidation by characteristic fragmentation in collision-induced dissociation (CID) [46,47], (3) improve the chromatographic separation performance and peak shape of analytes by adjusting retention time [48,49] and facilitate isomers separation by chiral derivatization reagents [50,51,52,53], (4) enhance detection sensitivity by the introduction of hydrophobic tags or permanent charged moiety to enhance ionization efficiency and improve the fragmentation pathway of analyte in CID for tandem MS analysis [54], and (5) reduce overall cost and improve throughout [55].

**Figure 2:**
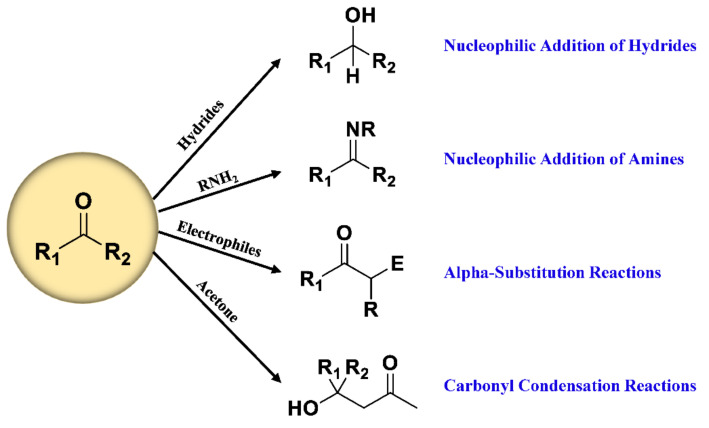
Classic reactions of carbonyl compounds.

**Figure 3:**
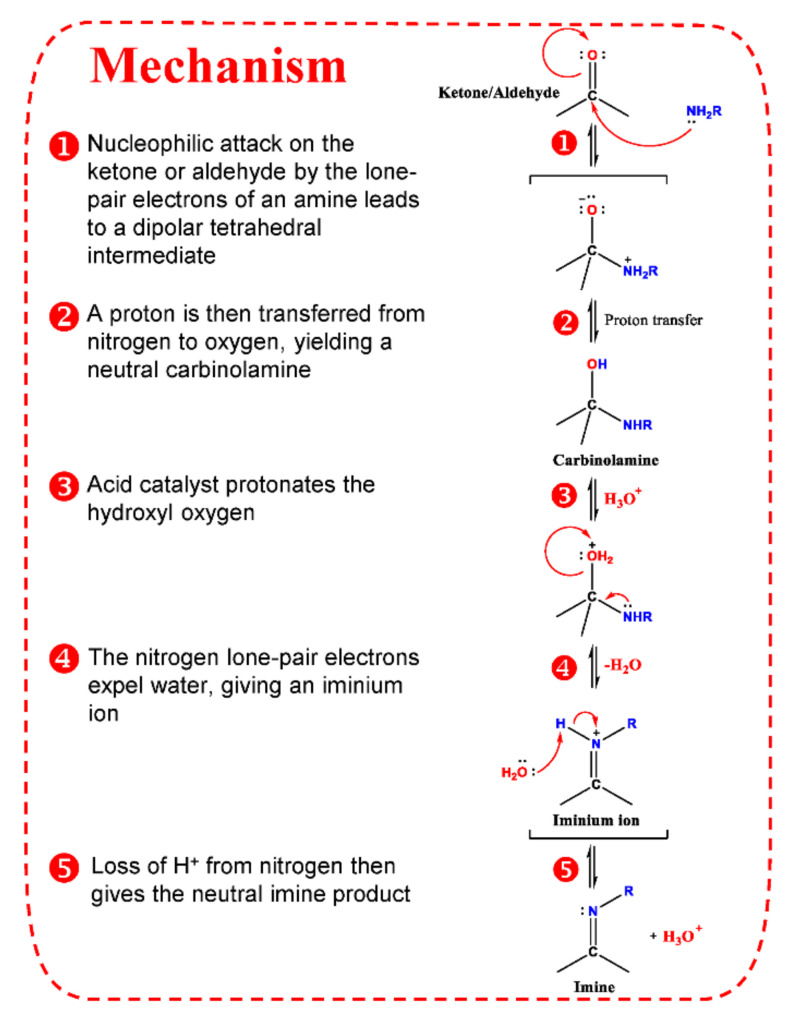
Mechanism of imine formation.

Therefore, many reagents have been developed based on classic carbonyl reactions (Figure 2) for detection of carbonyl-containing metabolites prior to LC-MS analysis. The essence of most used derivatization reagents are primary amines, which, as nucleophiles, react with carbonyl groups in the metabolites to yield imines. As illustrated in Figure 3, the rate-determining step of imine formation is the initiation of nucleophilic addition to yield a carbinolamine intermediate, which then loses water to give the imine. The substituents at the α-nucleophile moiety have a distinct effect on the stability of derivatives [56], and also determine the rate of derivatization reaction. Depending on the types of α-nucleophile moiety, derivatization reagent can be divided into four categories: hydrazines, hydrazides, hydroxylamines, and amines (Table S1).

### 2.1. Hydrazines

Hydrazine can react with carbonyl group to form hydrazones for better detection of carbonyl-containing metabolites. 2,4-Dinitrophenylhydrazine (DNPH) is the most widely used derivatization reagent, since it was first introduced [57,58]. Various DNPH labeling methods have been developed for the quantification of a wide range of carbonyl compounds, such as disinfection byproducts in water [59,60], toxins in food [61,62], harmful components in cigarette smoke [63], and photoproducts from solar-irradiated crude oil-seawater systems [64]. Chen et al. [65] were the first to optimize the DNPH derivatization condition in biological samples and established a rapid and sensitive isotope-dilution LC-MS/MS method for detection of urinary MDA with a limit of detection (LOD) of 1.6 pmol/mL^−1^. The derivatized MDA can be effectively retained on the C18 column and can be easily positively charged in the ESI ionization process.

Some carbonyl-containing compounds are highly reactive and easily degraded, such as α-keto acids. For these type of compounds, Zimmermann et al. [44] proposed a derivatization reagent, phenylhydrazine (PH), to stabilize these metabolites as soon as sample collection and prior to storage. PH can quench α-keto acids from further degradation and derivatization simultaneously at −20 °C, and therefore it is capable of absolute quantification of α-keto acids in biological fluids. The aromatic ring from PH added onto the α-keto acids can enhance the MS signal and increase the chromatographic retention times. The most challenging analysis of carbonyl-group containing metabolites is sugars with hemiacetal structure. Although carbohydrate derivatization takes longer to convert from the cyclic form to the open-chain form, the PH-derivatized sugars have a massive increase in sensitivity and, more importantly, they can produce a satisfied chromatographic resolution for isomers, such as glucose 6-phosphate and fructose 6-phosphate [37].

3-Nitrophenylhydrazine (3-NPH) is also an efficient derivatization reagent for quantitation of low-molecular weight (LMW) sugars [66]. Compared with other hydrazine-type derivatization reagents, there is no need to remove excessive 3-NPH in the reacted system as 3-NPH does not produce any background signals in negative ion ESI-MS detection mode, and therefore does not interfere with quantitation of LMW sugars. Han et. al. first used 3-NPH for quantitation of both LMW sugars and carboxylic metabolites [66,67]. Moreover, 3-NPH has been used to quantify flavor-active aldehydes, ketones, and organic acids in a wide concentration range up to six orders of magnitude [68].

Another derivatization reagent that has been used for both fatty aldehydes [69] and fatty acids [70] is 2,4-bis(diethylamino)-6-hydrazino-1,3,5-triazine (T3). The derivatization reaction of T3 with fatty aldehydes was quicker and milder than previously mentioned reagents, such as DNPH, owing to the high nucleophilicity of the hydrazine group in T3, and thus minimized the risk of fatty aldehydes degradation. In addition, derivatization with T3 significantly increased the sensitivity of fatty aldehydes due to the increased alkalinity of the derivatives, and also improved chromatographic performance due to the enhanced the hydrophobicity of the fatty aldehydes [69].

**Scheme 1:**
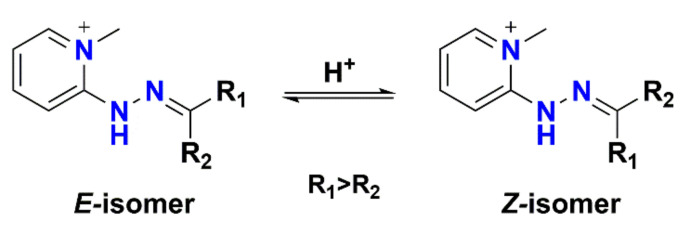
The *E-/Z*-isomers of HMP-derivatized carbonyl-containing metabolites.

Furthermore, 2-hydrazino-1-methylpyridine (HMP) and 2-hydrazinopyridine (2-HP) are also commonly used for derivatizing carbonyl-containing metabolites. Since HMP contains a fixed charged quaternary amine, derivatization with HMP can increase the detection sensitivity of cortisol and cortisone by 1000 times. Cortisol-bisHMP and cortisone-bisHMP are mainly formed after derivatization with HMP and each metabolite generates four peaks from four isomers (*E, E- E, Z- Z, E-* and *Z, Z-*isomers) in the chromatogram. Scheme 1 shows the *E/Z-*isomers of HMP-derivatized metabolites. To improve the detection sensitivity and quantitative accuracy, Magda et al. [71] modified the LC method to collapse all four peaks into one peak. Higashi et al. [72] proposed, for the first time, to enhance the sensitivity of ketosteroids through 2-HP derivatization. Later, this reagent was used for sensitive quantification of steroid hormones in human cerebrospinal fluid [73] and plasma [74]. Nadarajah et al. [75] tested five derivatization reagents (Girard’s reagent T (GRT), 1-benzyl-pyrrolidine-3-carboxylic acid hydrazide (1-BPH), 4-aminobenzoic hydrazide (4-ABH), isoniazid (INH), and 2-HP) for analysis of ketosteroids in saliva and 2-HP was superior not only in chromatographic behavior but also in signal response.

### 2.2. Hydrazides

Hydrazide reagents, such as Girard’s reagents and sulfonyl hydrazides, can react with a carbonyl group under weak acid conditions to form Schiff base. Girard’s reagents are types of hydrazide reagents with a quaternary ammonium group; Girard’s reagent T (GT) and Girard’s reagent P (GP) are used the most.

Dury et al. [76] proposed a highly sensitive and specific LC-MS/MS method for accurate quantitation of six neurosteroids based on a GT ketone-specific derivatization. Although six neurosteroids lack ionization efficiency in ESI-MS and are structurally highly similar, derivatization with GT successfully enhanced MS detection sensitivity and achieved better chromatographic separation. 4-Hydrazino-*N*,*N*,*N*-trimethyl-4-oxobutanaminium iodide (HTMOB) is a modified Girard’s reagent with three methylene linkages between the quaternary amine and hydrazine group. This modification strategy improved the ESI-MS/MS fragmentation characteristics of HTMOB derivatives. Thus, as compared with the GT derivatives, a 3.3- to 7.0-fold increase in the signal intensities of HTMOB derivatives was observed [77].

Similar to GT, GP introduces a fixed charge on a quaternary ammonium group, which relies on pyridinium moiety to increase ketosteroid solubility in reversed-phase solvents and give a strong response in positive mode ESI-MS detection [78]. Crick et al. [79] compared several modified Girard’s reagents and showed that the sensitivity of quaternary ammonium was similar to that of tertiary amine, but the quaternary ammonium aided the transfer of the positive charge from the pyridinium moiety to the steroid backbone, leading to the enhancement of the efficiency of MS^n^ fragmentation. In addition, derivatization of formylated nucleosides with GP exhibited the best detection sensitivity as compared with GT and 4-(2-(trimethylammonio)ethoxy)benzenaminium halide (4-APC) [80]. Later, Feng’s group synthesized four derivatization reagents (2-(2-hydrazinyl-2-oxoethyl)isoquinolin-2-ium bromide, HIQB; *N*,*N*,*N*-triethyl-2-hydrazinyl-2-oxoethanaminium bromide, THB; GT; GP) which all contained a quaternary ammonium group to enhance the detection sensitivity of carbonyl compounds. Among all the four reagents, HIQB labeling exhibited the highest hydrophobicity and the lowest LODs for almost all analytes. Moreover, the characterized fragment ions produced by the HIQB derivatives were higher in molecular weight and had a better MS response than those of other three reagents, so the HIQB derivatives could be less affected by the interference from low molecular weight metabolites. Taken together, HIQB has been chosen for profiling and quantification of carbonyl compounds using the stable isotope labeling double precursor ion scan MS strategy [81].

Unlike Girard’s reagents, 7-(diethylamino)coumarin-3-carbohydrazide (CHH) and “tandem mass tag hydrazine” (TMTH) contain a chargeable tertiary amino group which can also enhance the derivative signal in ESI-MS. The TMTH reagent combined with a carbonyl-reactive hydrazine group with tandem mass tags (TMT) was originally developed by Thomson et al. [82] and can yield characteristic mass reporter ions in an ESI-MS/MS analysis. Rigdova et al. [83] derivatized thirty oxosteroids with TMTH, with improved sensitivity that ranged from 14-fold to 2755-fold as compared with their underivatized steroids. CHH was the first derivatization reagent used for the simultaneous detection of both aliphatic and lipid-bound aldehydes and ketones to overcome their different properties [84]. The high hydrophobicity of CHH makes it possible to simultaneously extract water-soluble, lipid-bound, and highly hydrophobic aldehydes and ketones and prevent their loss during the extraction process. Moreover, characteristic neutral losses and fragment ions produced by the CHH derivatives during the CID process can be used to clearly identify the carbonyl compounds. In addition, Brkljacic et al. [85] designed a glutamic acid-related hydrazide reagent, which had higher sensitivity than DNPH owing to its higher ionization efficiency. Moreover, the fragmentations of glutamic acid-related hydrazones were structure specific and highly reproducible, which facilitated its use in sensitive MRM analysis.

Another type of hydrazide reagent is sulfonyl hydrazide, such as, *p*-toluenesulfonylhydrazine (TSH) and dansylhydrazine (Dns-Hz). TSH has been considered to be a suitable substitute for DNPH with similar reactivity. In addition, TSH can improve solubility of derivatives for better compatibility with aqueous biological samples. In addition, TSH has high volatility, which easily decomposes into gas-phase products at elevated temperatures, therefore, there is no risk of contamination of the MS by excessive TSH. However, the overall sensitivity of the analytes is reduced due to the formation of *E-* and *Z-* geometrical isomers of derivatives, which can be baseline separated in the UHPLC chromatograms. Some metabolites such as mercaptopyruvic acid significantly decrease during the derivatization process due to rapid oxidation and dimerization. Despite these limitations, TSH derivatization can distinguish a series of isomer pairs, for example, ketoleucine and ketoisoleucine, or acetone and propionaldehyde [86].

Dns-Hz is a fluorescent derivatization reagent, which has been widely used for fluorescence detection, and can also analyze the lipophilic reactive carbonyls (RCs) in biological samples by LC-ESI-MS/MS [87]. Compared with TSH, derivatization of RCs with Dns-Hz has several advantages. Most notably, the dimethylamino group of Dns-Hz, which is a tertiary amine, is easily protonated under acidic conditions, thereby, enhancing the ionization characteristics of RCs, and the Dns-Hz derivatives can produce a simple and clear product ion with an m/z value of 236.1 by CID. The drawback of Dns-Hz is that the capability of isomer separation may be relatively weak. For instance, the HNE and 4-hydroxy-2-hexenal (HHE), as a pair of stereoisomers, could not be separated in LC after derivatization.

### 2.3. Hydroxylamines

**Figure 4:**
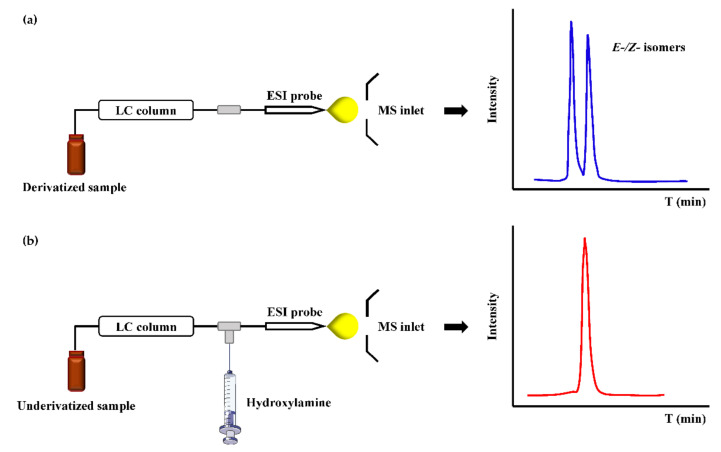
The scheme and chromatogram of oxime derivatives obtained by (**a**) pre-column derivatization and (**b**) post-column derivatization.

Unlike hydrazine-based reagents to form hydrazones, hydroxylamine (HA) reagents, including *O-*substituted hydroxylamine (-ONH_2_) and *N-*substituted hydroxylamine (-NHOH), can react with a carbonyl group to form oximes or nitrones. NH_2_OH is the simplest hydroxylamine reagent, and it has always been devoted to label ketosteroids. However, the poor hydrophobicity of NH_2_OH itself has limitation to improve the retention of metabolites. Liu et al. [88] quantified ten steroid hormones by hydroxylamine derivatization using HPLC-MS and a LOQ was achieved in the range of 0.05–5 ng/mL which was increased by 3.9–202.6 times after derivatization. Because hydroxylamine reacts fast and shows no contaminations to MS, Chen et al. [89] developed a fully automated in-tube SPME/LC-PCD-MS method, and demonstrated rapid and accurate quantification of urinary hexanal and heptanal by simple sample preparation and avoiding LC separation of *E-/Z-* isomers of oximes (Figure 4). The only catch is that the sensitivity needs to be further improved by employing more advanced mass spectrometers.

Apart from the poor hydrophobicity of NH_2_OH, it also suffers from producing complex metabolites resulting from Beckmann rearrangement reaction [90]. Derivatization with *O-*substituted hydroxylamine can overcome the rearrangement changes, which has attracted the attention of analytical chemists. Methoxyamine (MOA), which is similar to NH_2_OH, has also been dedicated to labeling steroid hormones containing carbonyl groups and has achieved detecting 29 steroid hormones at the level of pg/mL, which increased the sensitivity by three orders of magnitude after derivatization [91]. Owing to its poor retention, excessive methoxyamine can easily be diverted to waste without compromising the ionization efficiency of the derivative products [92].

*O-*(3-trimethylammonium propyl) hydroxylamine bromide is a quaternary aminooxy (QAO) reagent, which has an aminooxy (-ONH_2_) functional group as a carbonyl reactive group, and also processes a permanent positive charge, giving a better ionization efficiency in ESI-MS. As compared with HA, the signal-to-noise (S/N) ratio of QAO reagent for testosterone analysis is about 10 times higher than that of HA [93]. Furthermore, the use of QAO reagent can improve the reliability of hormone analysis in the complex matrix due to the formation of chromatographically separated derivative isomers [94].

*N-*[2-(aminooxy)ethyl]-*N,N-*dimethyl-1-dodecylammonium (QDA) is also a quaternary ammonium tag, which can greatly improve detection sensitivity of ESI-MS. Meanwhile, QDA has a hydrophobic domain for better retention on column [95]. The key principle of this reagent is based on the use of *N,N-*dimethyl-*p-*phenylenediamine as a catalyst to accelerate the derivatization reaction and, subsequently, the quench reaction at -80 °C. This method can minimize the degradation of unstable metabolites [96].

A brominated *O-*substituted hydroxylamine, namely 1-((ammoniooxy)methyl)-2-bromobenzene chloride (BBHA) has been selected as a new probe for a mild and rapid derivatization of carbonyl compounds [97]. On the basis of the existence of the characteristic ^79^Br/^81^Br isotopic pattern, carbonyl compounds can be specifically detected in complex mixtures. Different modification groups on the *O-*substituted hydroxylamine can achieve different detection purposes. For example, aminooxy-*N-*(3-perfluorooctyl-propyl) acetamide [98] is a fluorous *O-*substituted hydroxylamine tag and can selectively react with carbonyls to yield oximes, which can be extracted from the biological fluids using a fluorine spin column to simplify analysis. In addition, the fluorous *O-*substituted hydroxylamine tag can increase the hydrophobicity of the analytes, achieving a 30-fold increase in sensitivity. The method achieved both chromatographic separations and MS/MS detection of the structural isomers of acetoacetate and hydroxybutyrate.

Conway et al. [99] proposed a new strategy which used a chemoselective probe immobilized to magnetic beads to efficiently separate carbonyl metabolites from biological matrix, thereby, reducing MS background interference and enhancing sensitivity by up to six orders of magnitude. In addition, the biorthogonal cleavage of the chemoselective probe can be cleaved from the magnetic beads under mild conditions to avoid degradation of labile carbonyl metabolites [100].

Although the chromatographically separated *E-/Z-* isomers formed by the reaction of asymmetric carbonyl metabolites with *O-*substituted hydroxylamines have been considered useful for structure identification in some cases, it brings huge challenges to MS sensitivity. Guan et al. [101] firstly designed and synthesized two novel *N-*substituted coumaroyl hydroxylamines (4-hydroxylaminopropyl-7-methoxylcoumarin (HAMC) and 7-hydroxy-4-(4-(hydroxyamino)butyl)-2*H*-chromen-2-one (HAHC)) with long aliphatic chain, which enhanced the ionization efficiency of derivatives. Compared with other *O-*substituted hydroxylamines, HAMC and HAHC provided a mild and efficient labeling in less than 30 min at room temperature, and also overcame the shortcomings of forming *E-/Z-* isomers. These novel *N-*substituted hydroxylamines have been applied for the determination of furfuraldehydes in foodstuffs, and their further applications on the analysis of more aliphatic aldehydes are promising [102].

### 2.4. Amines

Amines are another type of derivatization reagent for carbonyl metabolites, which are usually used in combination with a reducing reagent, such as sodium cyanoborohydride (NaBH_3_CN) and 2-picoline borane (pic-BH_3_), to reduce the Schiff base derivative to a single, more stable secondary amine product. Since many ketones tend to react more slowly than aldehydes [103], amines are often used to derivatize aldehydes. The reaction can proceed completely to prevent hydrolysis, and hence the method allows samples to be stored over longer periods.

Aniline has been used to react with sugars for better detection for many years [104]. The derivatization reaction of sugars should be carried out at a low pH since it promotes the opening of the sugar ring, while aniline is a weak base that can still have enough unprotonated form to react with sugar at a low pH. As a derivatization reagent, aniline can achieve the best separation for structural isomers such as four common hexoses (glucose, fructose, mannose, and galactose) [105] and also enables targeting of multiple functional groups that are more suitable for global metabolic profiling [106].

3-Amino-9-ethylcarbazole (AEC) has been used to derivatize sugar phosphates in biological fluids based on reductive amination [107]. However, the derivatization reaction for biological samples, often in the liquid phase, is usually accompanied by shortcomings, such as time-consuming, excessive derivatization reagents and low derivatization efficiency. Qin et al. [108] modified the method by firstly employing SiO_2_@PD_Ti^4+^ microspheres to selectively enrich sugar phosphates and remove the excess number of other metabolites prior to derivatization, and then react with AEC based on solid-phase derivatization. This modification has significantly shortened derivatization time and reduced the interference of the residual derivatization reagents on the ionization efficiency, leading to three orders of magnitude improvement on the sensitivity for detection of sugar phosphates and making it possible to determine trace sugar phosphates in small amounts of biological fluids.

**Scheme 2:**
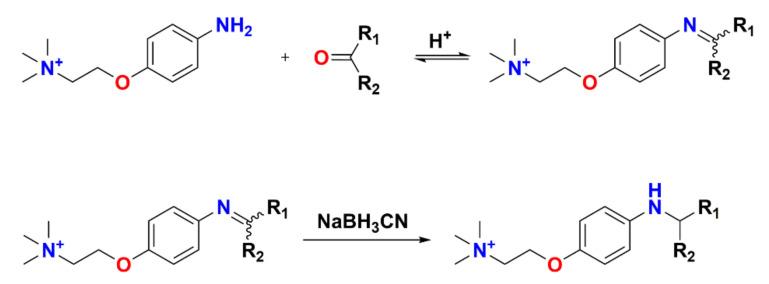
Pathway for imine formation and reduction using aldehydes and 4-APC.

Unlike aniline, 4-(2-(trimethylammonio) ethoxy) benzenaminium halide (4-APC) is a selective derivatization reagent for the analysis of aldehydes [109]. The nucleophilic group of 4-APC has aniline moiety and its pKa is low enough to retain nucleophilic property to ensure fast reduction of the resulting imine by NaBH_3_CN, as shown in Scheme 2. Due to the difficulty in the formation of the initial imine between the ketone and the aniline moiety, 4-APC can selectively label aldehydes. In addition, a quaternary tetraalkylammonium group can connect to the para position of the aniline through an ethoxy spacer to provide a permanent positive charge for enhancing MS sensitivity, while minimizing the induction of the nucleophilicity of NH_2_ by the positive charge. Labeling with 4-APC can dramatically increase the MS sensitivity of aldehydes, such as hexanal, by more than 1000 times [110]. Another derivatization reagent, 4-(2-((4-bromophenethyl)dimethylammonio)ethoxy)benzenaminium dibromide (4-APEBA), has been designed based on 4-APC to address some additional stringent issues in a metabolite analysis, such as biomarker analysis of lipid peroxidation [111]. 4-APEBA contains a bromophenethyl group which can significantly enhance the recognition of metabolites through the characteristic ^79^Br/^81^Br isotopic pattern. Meanwhile, the molecular weight of 4-APEBA is higher than that of 4-APC, resulting in a higher S/N ratio of the 4-APEBA derivatives in biological fluids.

Sun et al. [112] developed a new derivatization reagent, 4-(1-methyl-1*H*-phenanthro [9,10-*d*]imidazol-2-yl)phenlamine (MPIA). MPIA can label aldehydes through reductive amination under weak acid condition to produce secondary amine derivatives without *E-/Z-* isomers and few byproducts. As compared with the widely used DNPH, MPIA derivatives show higher sensitivity, which is probably attributed to the strong proton affinity of the lone pairs on imidazole nitrogen atom and the high hydrophobicity of MPIA derivatives.

Different from monoamines, diamines react with α-dicarbonyl compounds to form cyclic compounds, so that a single derivatized product can be obtained without a reducing reagent.

It is well known that *o-*phenylenediamine (OPD) is the most commonly used derivatization reagent for quantitative α-dicarbonyl compounds [113,114,115,116]. OPD reacts with α-dicarbonyl compounds, such as methylglyoxal, to produce stable 2-methylquinoxaline with high MS detection sensitivity [117]. However, when it is used to determine the content of α-dicarbonyl compounds in biological samples with high levels of sugar, such as honey, OPD may undergo Maillard reaction with reducing sugars to form α-dicarbonyl compounds, thereby, affecting quantification. Hurtado-Sanchez et al. [118] compared three derivatization reagents (2,4,5-triamine-6-hydroxypyrimidine (TRI), 5,6-diamino-2,4-hydroxypyrimidine (DDP), and OPD) and pointed out that TRI was the most advantageous derivatization reagent for analyzing α-dicarbonyl compounds in samples with high sugar content, due to much less amount of interfering α-dicarbonyl compounds formed as compared with DDP and OPD.

Similarly, 3,4-diaminobenzophenone (DABP) has been proven to have better performance in determining the levels of malondialdehyde in biological fluids [119]. DABP can generate a stable derivative without using reducing agent, hence, less interference. DABP derivatives can achieve approximately 50–100 times higher in MS sensitivity as comparing with DNPH, and therefore no concentration step is required when directly determining the levels of malondialdehyde in urine and saliva samples.

### 2.5. Others

Other derivatization reagents containing unique functional groups have been employed for the detection of carbonyl compounds. According to the Schiff reaction of aldehydes with D-cysteine to form only a stable substituted thiazolidine carboxylic acids, Kim et al. [120] developed a simple and rapid method to determine eight aldehydes in beverages and avoid the drawback of hydrazine as derivatization reagents forming syn- and anti-isomers. Acetylacetone is a specific derivatization reagent to formaldehyde according to the principle of the Hantzach reaction [121]. As compared with the commonly used DNPH, the derivatization with acetylacetone takes place under mild conditions and forms stable derivatives, but it requires a longer reaction time of about 30 min. Acetylacetone has also been used to detect trace levels of free formaldehyde in cosmetics by LC-PCD-UV with limits of detection and quantification levels of 0.7 and 2.3 ng/mL^−1^ [122]. However, acetylacetone is more toxic and has weak fluorescence, which is not easy to store, which has prevented it from common use.

Cyclohexane-1,3-dione (CHD) is an alternative derivatization reagent since it is less toxic and irritant than acetylacetone and other derivatization reagents, such as 3-methyl-2-benzothiazolinone hydrazone (MBTH) and phenylhydrazine-4-sulfonic acid (PHSA) [123]. The derivatization using CHD has high selectivity and sensitivity without the need of pH adjustment, and each different aldehyde only forms one derivative without the formation of isomers. Moreover, the dimethyl CHD forms better derivatives with aldehydes due to their increased hydrophobicity [124].

Similar to acetylacetone, ammonium sulfite has also been used for post-column derivatization of aldehydes [125]. Ammonium sulfite can react with aldehydes to perform LC-MS analysis in negative ionization mode with relatively strong anti-interference ability. In addition, the characteristic fragments of the derivative can be obtained with extremely low collision energy, resulting in a significant reduction in false positive detections in mass spectrometry. Thus, compared with the LC-PCD-MS method that employs HA to react with aldehydes [89], ammonium sulfite derivatives suffer less matrix effects and can improve detection sensitivity by five-fold.

9,10-Phenanthrenequinone (PQ) has been selected as a derivatization reagent for aliphatic aldehydes and the benefit of this methods is the high selectivity for aliphatic aldehydes, hence, significantly reducing interferences from other carbonyl compounds including ketones and carboxylic acids [126]. The high selectivity of PQ enables the detection sensitivity of aliphatic aldehydes in the range of 4–100 pM, which is about 19–1000-fold higher than can be achieved by MPIA. In addition, PQ is commercially available, and hence can save the time to synthesize a derivatization agent, which is an overall advantage over previously mentioned reagents, for example, CHD-based reagents and MPIA.

*N*-(1-chloroalkyl)pyridinium quaternization (NAPIQ) is also a new derivatization method for labeling aliphatic aldehydes [127]. Unlike common precharged reagents, uncharged pyridine and thionyl chloride in NAPIQ are designed to add a permanently charged label to the aldehyde. Pyridine is far less competitive in ionization than charged derivatives, whereas excess thionyl chloride can be easily removed with deionized water, which converts thionyl chloride into less residual sulfur dioxide bubbles. Therefore, this method could detect aliphatic aldehydes directly after derivatization without further postprocessing. Moreover, NAPIQ has been designed to label ketosteroids with alcoholic or α,β-unsaturated ketone moieties and carbohydrates by changing the reaction solvent [128]. This simple derivatization method only generates one product and is suitable for ketosteroids containing more than one carbonyl groups. Thus, the sensitivity and selectivity of the NAPIQ strategy are higher than those of Girard reagents.

Carbohydrates are a complex class of metabolites, clearly, one method will not be adequate to detect all carbohydrates. Simultaneous quantitation of sixteen monosaccharides through 1-phenyl-3-methyl-5-pyrazolone (PMP) derivatization could achieve baseline separation of isomers within 10 min by taking advantage of the high hydrophobicity of PMP molecules [129]. Derivatization of xylose and arabinose with 3-NPH could not achieve effective separation of the two metabolites, however, they could be effectively separated using PMP derivatization. Owing to efficient separation, the LODs of most monosaccharide derivatives with PMP could reach ranges between femtomoles and attomoles, which was increased by more than 1000-fold as compared with picomoles obtained by UV or ion trap MS.

## 3. Application of the Stable Isotope-Coded Derivatization Method in LC-MS Analysis of Carbonyl-Containing Metabolites

Although modification of carbonyl-containing metabolites by a chemical derivatization strategy can overcome the obstacles of detectability and sensitivity, it is still insufficient with an accuracy issue due to instrument drift and matrix effects produced by the co-eluting components. The emergence of the ICD strategy provides a good solution to the problems encountered above. Thus, LC-MS methods that utilize ICD has been applied in metabolomics including quantitative and profiling analysis (Table 1).

### 3.1. Absolute Quantification

**Figure 5:**
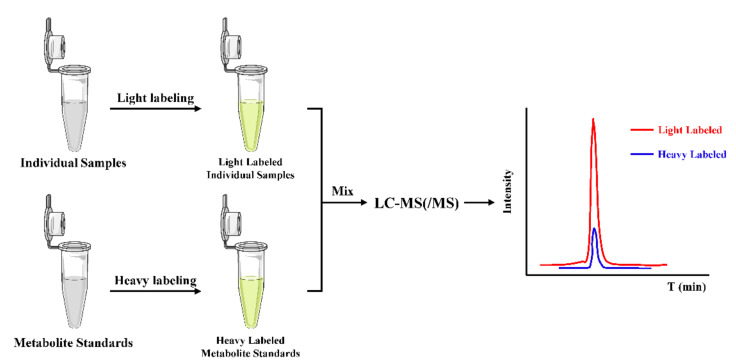
General workflow of the ICD strategy for the absolute quantification.

In the ICD strategy, as illustrated in Figure 5, the stable isotope-coded moiety of the derivatization reagent is used to label the standard metabolites of known amounts to form derivatives, which acts as ISs and can generate isotope ISs for all the target metabolites simultaneously in the derivatization step. Therefore, ICD could significantly improve the quantitative accuracy and precision by compensating for ESI matrix effects and instrument drift.

As illustrated in Figure 5, the HMP/d_3_-HMP reagent pair have been utilized for the differentiation and quantification of levels of neurosteroids in brain in response to immobilization stress and antipsychotic drug administration [130]. The HMP derivatization has improved the detection sensitive by 500- and 3000-fold for allopregnanolone and pregnenolone and the use of d_3_-HMP can significantly reduce the run-to-run ionization differences and achieve high reproducibility and accuracy. However, the isotope effect may cause derivatives and their isotope ISs to be eluted at a different time in chromatographic separation. The shift in retention time may be great enough to cause a different matrix effect in LC-MS, especially when using electrospray ionization (ESI). Due to the deuterium isotope substitution, the d_3_-HMP derivatives always eluted about 0.1 min earlier than the HMP derivatives and showed a slightly undesirable effect on the quantitative analysis.

**Scheme 3:**
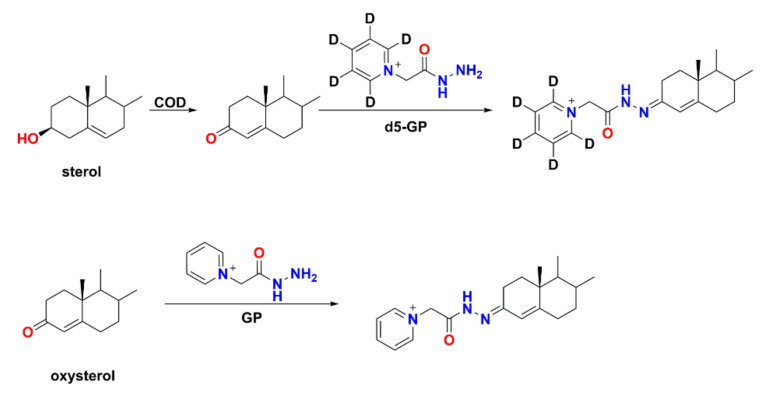
Reaction strategy for simultaneous quantification of sterol and oxysterol.

The GP/d_5_-GP reagent pair have been used to label steroid hormones in follicular fluid samples and steroid hormone standards derivatized with d_5_-GP act as internal standards for accurately quantifying steroid hormones by calibrating the variation of MS response due to the ion suppression and matrix effects [131]. GP/d_5_-GP derivatives also accomplish a significant improvement in the ionization efficiency by introducing a permanently charged moiety. The mass difference between d_5_-GP and GP is 5 Da, which minimizes the negative effects of naturally occurring stable isotopes, such as ^13^C. In addition, deuterium is labeled on the ring of d_5_-GP, hence, minimizing the H/D exchange, and providing great stability as molecular tag. The GP/d_5_-GP reagent pair have been used to simultaneously determine the levels of aldosterone in the left and right adrenal venous serum samples in a single run [136]. This approach can improve the reliability of the quantification and in the meantime shortened the analysis time, hence, enhancing the throughput of the analysis. Since GP can label sterols with a carbonyl group, the sterols with a hydroxyl group and oxysterols with a carbonyl group cannot be analyzed in a single LC-MS analysis using GP derivatization. However, GP/d_5_-GP can be used to quantify oxysterols and sterols simultaneously if sterols are oxidized by cholesterol oxidase (COD) first to contain a carbonyl group (Scheme 3) [137]. On the basis of this principle, it is possible to quantify up to four different samples in a single run by using differential mass tags and isobaric mass tags simultaneously through multiplex methods [138].

GP has been used to derivatize ketosterols, such as 7α-hydroxy-4-cholesten-3-one (7αC4), to form hydrazones; however, the application of this method is limited by low sensitivity as the LOQ is close to the maximum concentration in the circulation system [139]. The detection sensitivity of QAO derivatives is at least 2- to 3-fold higher than that of GP, therefore, De Barber et al. [132] utilized QAO and d_3_-QAO reagents to achieve sensitive isotope dilution quantification of 7αC4 and 7α, 12α-dihydroxy-4-cholesten-3-one (7α12αC4) in the plasma of cerebrotendinous xanthomatosis (CTX) patients. As compared with the GP derivatives, the equilibrium constant of oxime is higher, thereby, displaying a better stability of QAO derivatives at room temperature. As comparing with the commercially available QAO-d_0_-tagged ketosterol-d_7_ internal standard, QAO-d_3_-tagged ketosterol-d_0_ internal standard showed acceptable correlation. Thus, the isotope-coded derivatization reagent, d_3_-QAO, could be used to generate internal standard for quantification. Later, the method has been modified to reduce the analysis time by two minutes for each sample [140].

Among the developed derivatization reagents, DNPH is the most popular derivatization reagent for aldehyde, but for absolute quantification purposes, its isotope-coded derivatization reagents, d_3_-DNPH or ^15^N_4_-DNPH, are expensive and difficult to prepare. In this case, MPIA/d_3_-MPIA has been developed, which can be economically and simply prepared with low-cost d_0_/d_3_-iodomethane (CH_3_I/CD_3_I). Moreover, d_0_/d_3_-MPIA, as compared with DNPH, exhibits higher sensitivity with mild derivatization conditions for the quantification of aliphatic aldehydes in aquatic products [112].

Considering the deuterium isotope effect on the quantification, the use of ^13^C/^15^N isotope could avoid potential retention time variations induced by deuterium labeling. Sobsey et al. [133] developed a new method for quantification of malondialdehyde using 3-NPH as the derivatization reagent, which has achieved enhanced sensitivity and increased the linearity range with shorter analysis time than the previous DNPH-LC-UV method. While using ^13^C_6_-3-NPH to generate an internal standard (^13^C_6_-3-NPH-MDA), the quantitative accuracy and precision of MDA in human plasma can be achieved without interference from matrix effect.

In addition, ^14^N/^15^N-ammonium acetate coupled with PQ is the first commercially available ICD reagent pair, which avoids the complicated and time-consuming synthesis process. EI-Maghrabey et al. [134] used this cost-effective ICD reagent and developed a method with very sensitivity and high accuracy to determine two highly reactive 4-hydroxy-2-alkenals, i.e., HHE and HNE, in human serum at ultratrace levels. The LOD for both HHE and HNE of the method is about 20–32000 and 4–8700 times lower than all the reported methods. The benefit of using ^14^N/^15^N as isotopic signature is that it can completely avoid the deuterium isotope effect, resulting in effectively minimized matrix effects and increased sensitivity during LC-ESI-MS/MS analysis.

There are some other commercially available ICD reagents, such as d_0_/d_8_-acetylacetone [121], d_0_/d_5_-pyridine, and aniline/^13^C_6_-aniline. Formaldehyde has been derivatized to 3,5-diacetyl-1,4-dihydrolutidine (DDL) and d_12_-DDL by d_0_/d_8_-acetylacetone for its accurate quantification in personal-care products [121]. In addition, *N*-(1-chloroalkyl)pyridinium quaternization has been developed to identify and quantify aliphatic aldehydes in human thyroid carcinoma and para-carcinoma tissue [127]. Pyridine-d_5_ labeled aliphatic aldehydes have been prepared as internal standards. Furthermore, d_0_/d_5_-pyridine could also be used to simultaneously label different functional groups including hydroxyls and carbonyls [141]. Similarly, aniline/^13^C_6_-aniline could also label multiple functional groups to simultaneously quantify metabolites involved in central carbon and energy metabolism [104,106].

### 3.2. Submetabolome Profiling

Another important application of the ICD strategy is submetabolome profiling. By using the ICD reagent pair, carbonyl-containing metabolites will exhibit a pair of peaks with equal intensity in MS or MS/MS spectra, which can be easily recognized in a mixture.

Dns-Hz/^13^C_2_-Dns-Hz [36] is a typical ICD reagent pair for labeling carbonyl for submetabolome profiling and has been used to profile the carbonyl-containing metabolites in human urine, which detected 2030 ± 39 pairs per run with high reproducibility. This approach has been demonstrated to be valuable for profiling important carbonyl-containing metabolites in complex biological fluids. This ICD reagent combined with other labeling reagent could achieve a broader coverage of carbonyl, amine, carboxyl, and hydroxyl submetabolomes [142].

QDA/^13^CD_3_-QDA is another ICD reagent pair for profiling carbonyl-containing metabolites in tissue and cell extracts [95]. The hydrophobic moiety of QDA provides better retention property for carbonyl-containing metabolites, which is capable of removal interferences from hydrophilic metabolites, while the stable isotope signatures enable simple identification of carbonyl metabolites. This method is suitable for quantification of low abundance carbonyl metabolites [96].

Another example of ICD reagent is d_0_/d_20_-2,4-bis(diethylamino)-6-hydrazino-1,3,5-triazine (T3/D3) [69], which has been utilized for both the discovery and comprehensive characterization of endogenous fatty aldehydes in rat plasma and brain tissue. A total of 43 and 19 fatty aldehydes were significantly changed in plasma and brain tissues between the control group and the model group, which revealed the capability of T3/D3 in profiling of fatty aldehydes.

4-APC/d_4_-4-APC ICD pairs has also been used to profile aldehyde-containing compounds [110]. Since 4-APC and d_4_-4-APC could produce two characteristic neutral fragments, 87 Da and 91 Da, double neutral loss scan (DNLS) could be performed for identification of potential aldehyde-containing compounds. The DNLS has inherent high selectivity as compared with the full scan mode, therefore, this method can improve the detection sensitivity. This reagent, coupled with black phosphorous, as MALDI matrix, exhibits strong capability in the quantitative analysis of aldehydes in complex samples including saliva, urine, and serum [143]. 4-APC/d_4_-4-APC has also been used in combination with BQB/d_7_-BQB for simultaneous profiling of aldehydes and thiols in beer samples [144].

**Figure 6:**
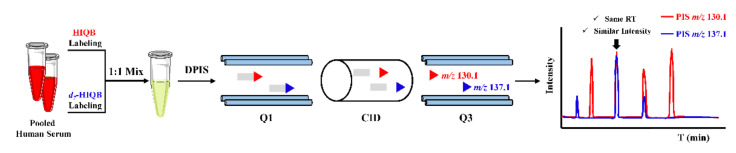
The scheme for profiling of carbonyl compounds using HIQB/d_7_-HIQB.

Stable isotope labeling double precursor ion scan MS analysis (IL-DPIS-MS) strategy is an alternative method for untargeted metabolic profiling without HRMS or prior knowledge of a metabolites list. It was firstly proposed by Feng’s group, in 2017, for profiling and relative quantitation of carbonyl compounds in human serum derivatized with HIQB/d_7_-HIQB [81]. As shown in Figure 6, two characteristic product ions of *m/z* 130.1 and 137.1 were obtained from HIQB/d_7_-HIQB labeled carbonyl compounds under CID and were utilized for double precursor ion scans to characterize carbonyl compounds, which significantly improved the detection selectivity and accuracy as compared with the full scan mode. A total of 44 carbonyl compounds could be identified in serum. These detected metabolites could differentiate myelogenous leukemia (ML) patients from healthy controls, which could serve as biomarkers for ML. Moreover, aberrant serum carbonyl compound profiles can also reflect poor cognitive performance of subclinical carotid atherosclerosis (SCA) patients [13].

**Figure 7:**
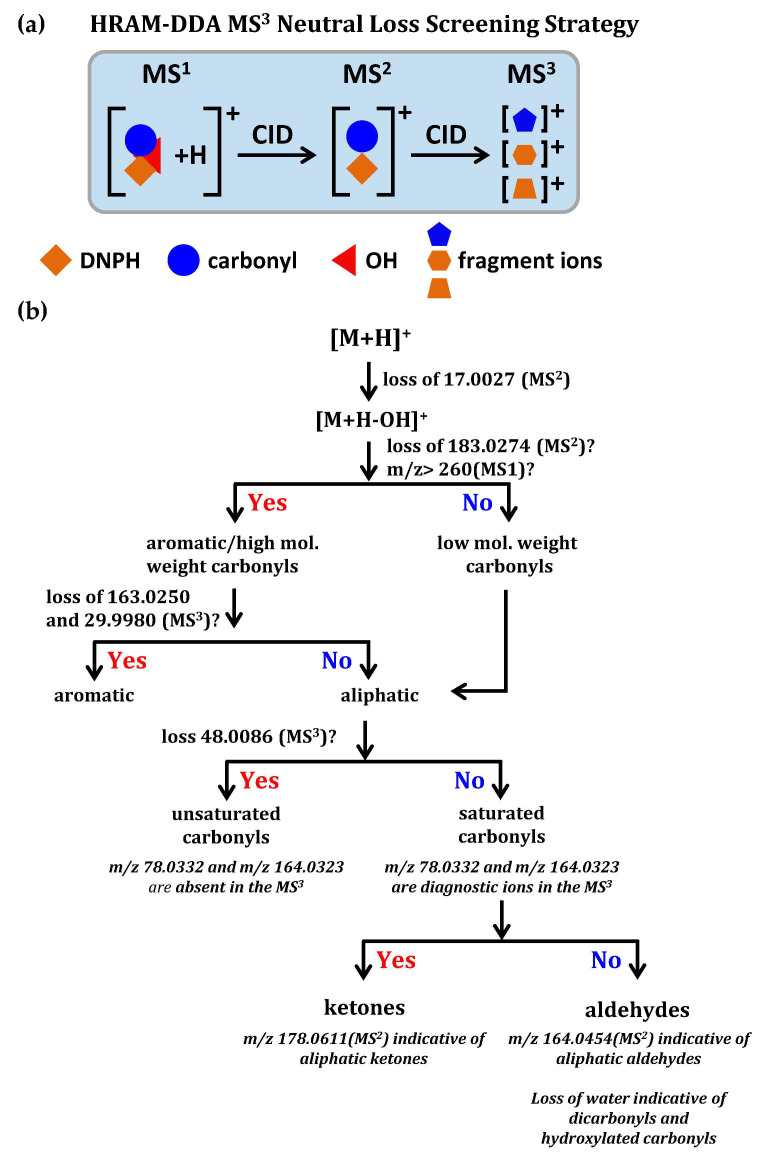
(**a**) Scheme of HRAM-DDA MS^3^ neutral loss screening strategy; (**b**) classification scheme for characterizing known and unknown DNPH-derivatized carbonyls. Adapted with permission from [135]. Copyright 2017 American Chemical Society.

Dator et al. [135] firstly used the high resolution accurate mass (HRAM) screening strategy combined with diagnostic neutral loss of hydroxyl radical triggering MS^3^ fragmentation for characterizing known and unknown DNPH-derivatized carbonyls, as shown in Figure 7. The HRAM screening strategy has reliably determined the neutral loss of DNPH-derivatized carbonyls, which was due to the loss of hydroxyl radical (17.0027 Da), rather than other interferences, such as NH_3_ (17.0266 Da). Observation of the neutral loss of hydroxyl radical triggered MS^3^ fragmentation to produce characteristic ions (m/z 78.0332 and 164.0323) in the MS^3^, which minimized false positive identification. In addition, this method coupled with d_3_-DNPH allowed relative quantification of carbonyl compounds in saliva [135].

## 4. Conclusions

Carbonyl-containing metabolites widely exist in biological samples and have important physiological and pathological functions. Thus, quantitatively accurate analysis of carbonyl-containing metabolites with high sensitivity is crucial to provide insight into metabolic pathways as well as disease mechanisms. However, quantitative analysis remains a challenge due to the high diversity of physicochemical properties, and the high dynamic concentration range of carbonyl-containing metabolites presented in variations of sample matrix. Although RPLC-ESI-MS is widely used for the measurement of these metabolites, it is still challenging to directly analyze carbonyl-containing metabolites using RPLC-ESI-MS due to poor ionization efficiency of neutral carbonyl groups in ESI. In this regard, modification of carbonyl-containing metabolites by a chemical derivatization strategy can convert them into MS-detectable and LC-separable derivatives. Almost all the derivatization reagents are primary amines, which, as nucleophiles, react with carbonyl groups in the metabolites to yield imines. Since substituents at the α-nucleophile moiety having a distinct effect on the stability of derivatives, the types of derivatization reagents play an important role in the rate of derivatization reaction, which can be divided into four categories: hydrazines, hydrazides, hydroxylamines, and amines. However, a pair of *E-/Z-* isomers can be formed as derivatization products and exhibit broad peaks or be separated in the LC chromatograms, which obstruct quantification. To overcome this obstacle, a post-column derivatization strategy or reductive amination method using a reducing agent could be an efficient solution. Although modification of carbonyl-containing metabolites by a chemical derivatization strategy can overcome the obstacles of detectability and sensitivity, it is still insufficient to achieve accurate quantification, since instrument drift and matrix effects, produced by the co-eluting components, result in fluctuations of ionization efficiency of metabolites in ESI. The emergence of ICD provides a good solution to the problems encountered above. Thus, LC-MS methods that utilize ICD have been applied in metabolomics including quantitative and profiling analysis. In addition, ICD makes multiplex isobaric or differential mass tags possible, which enables multiple samples to be detected in a single run, whereby reducing instrument running time, and also avoids the variation of MS response due to instrument drift. A simultaneous ICD strategy for multichannel submetabolome analysis has several unbeatable advantages, including high coverage of metabolites and quantitative high throughput, which makes molecular phenotyping of a population possible.

## Figures and Tables

**Table 1:** Summary of ICD reagents for carbonyl-containing metabolites and their applications.

Reagent	ICD Reagent	Analytes	Matrix	Application	Year & Ref.
HMP	d_3_-HMP	Brain neurosteroids	Rat brain	Differential and quantitative analysis of the changes in the brain neurosteroids levels by immobilization stress and antipsychotic drug administration	2016 [130]
GP	d_5_-GP	Steroid hormones	Human follicular fluid	Quantitative analysis of androgenic and progestagenic steroids in polycystic ovarian syndrome patients compared with healthy controls	2016 [131]
QAO	d_3_-QAO	Ketosterols	Human plasma and dried blood spots	Sensitive isotope dilution quantification of 7αC4 and 7α12αC4 between CTX and unaffected samples	2014 [132]
MOA	d_3_-MOA	Ketosteroids hormones	Meat	Sensitive and accurate quantification of ketosteroids hormones in meat using MRM scan mode	2015 [92]
3-NPH	^13^C_6_-3-NPH	MDA	Human plasma	Quantification of MDA in major depressive disorder patients	2016 [133]
MPIA	d_3_-MPIA	Aliphatic aldehydes	Aquatic products	Quantification of aliphatic aldehydes in aquatic products	2014 [112]
Ammonium acetate and PQ	^15^N-ammonium acetate and PQ	HNE & HHE	Human serum	Simultaneous determination of HNE and HHE in the serum of healthy controls and pathological conditions	2018 [134]
Acetylacetone	d_8_-acetylacetone	Formaldehyde	Personal-care products	Accurate quantification of formaldehyde in children’s personal-care products	2017 [121]
Pyridine	d_5_-pyridine	Fatty aldehydes	Human thyroid carcinoma and para-carcinoma tissue	Identification and quantification of free fatty aldehydes in the human thyroid tissue	2016 [127]
Aniline	^13^C_6_-aniline	Carbonyl-containing metabolites	Cell-free protein synthesis	Absolute quantification of cell-free protein synthesis metabolism	2019 [104]
Dns-Hz	^13^C_2_-Dns-Hz	Carbonyl submetabolome	Human urine	Profiling the carbonyl submetabolome in human urine	2017 [36]
QDA	^13^CD_3_-QDA	Carbonyl metabolites	Tissue and cells extraction	Profiling and quantification of carbonyl metabolites	2012 [95]
T3	D3	Fatty aldehydes	Rat plasma and brain tissue	Discovery and comprehensive characterization of endogenous fatty aldehydes in rat plasma and brain tissue	2016 [69]
4-APC	d_4_-4-APC	Aldehydes	Human urine and white wine	Identification of potential aldehyde-containing compounds in human urine and white wine	2015 [110]
HIQB	d_7_-HIQB	Carbonyl compounds	Human serum	Profiling and quantitation of carbonyl compounds in human serum between myelogenous leukemia patients and healthy controls	2017 [81]
DNPH	d_3_-DNPH	Carbonyl compounds	Saliva	Simultaneous identification and relative quantification of carbonyls in human biological fluids	2017 [135]

## Supplementary Materials

The following are available online, Table S1: Derivatization reagents for carbonyl-containing metabolites.
