# Supplementary material for: Progress and Challenges in Quantifying Carbonyl-Metabolomic Phenomes with LC-MS/MS
Source: Molecules. 2021 Oct 12;26(20):6147. doi: 10.3390/molecules26206147 (PMC8541004; doi:10.3390/molecules26206147)
Supplement: Supplementary file 1 [file molecules-26-06147-s001.zip › molecules-1390599-supplementary.pdf]

molecules

MDPI

Supplementary Information

Review

Progress and Challenges in Quantifying Carbonyl-Metabolomic Phenomes with LC-MS/MS

Yuting Sun [1,2,3], Huiru Tang [3] and Yulan Wang [4,*]

[1] Key Laboratory of Magnetic Resonance in Biological Systems, State Key Laboratory of Magnetic Resonance and Atomic and Molecular Physics, National Centre for Magnetic Resonance in Wuhan, Wuhan Institute of Physics and Mathematics, Innovation Academy for Precision Measurement Science and Technology, Chinese Academy of Sciences, Wuhan 430071, China

[2] University of Chinese Academy of Sciences, Beijing 10049, China

[3] State Key Laboratory of Genetic Engineering, Zhongshan Hospital and School of Life Sciences, Human Phenome Institute, Metabonomics and Systems Biology Laboratory at Shanghai International Centre for Molecular Phenomics, Fudan University, Shanghai 200438, China

[4] Singapore Phenome Centre, Lee Kong Chian School of Medicine, Nanyang Technological University, Singapore 639798, Singapore

* Correspondence: yulan.wang@ntu.edu.sg; Tel.: +65-69041106

Contents

Table S1: Derivatization reagents for carbonyl-containing metabolites.

Table S1. Derivatization reagents for carbonyl-containing metabolites

Reagents	Structure	Analytes	Derivatization conditions	Comments	Year& Ref.
	**Hydrazines**			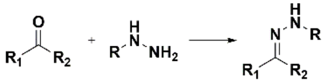	
DNPH	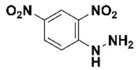	MDA	FA in ACN/water (1:19), 37 °C, 70 min	The first to optimize the DNPH-derived conditions of urine samples	2011 [65]
PH	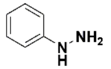	α-Keto Acids	In ACN/MeOH/water (2:2:1), -20 °C, 1 h	Simultaneous quenching and derivatization at -20 °C to avoid further degradation	2014 [44]
3-NPH	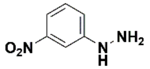	13 mono- and disaccharides	EDC and pyridine in MeOH/water (3:1), 50 °C, 1 h	The first method to simultaneously quantify LMW sugars and organic acids using a single derivatization reagent	2016 [66]
T3	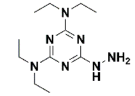	Fatty aldehydes	FA in MeOH, 37 °C, 15 min	Quicker and milder than previously mentioned reagents	2016 [69]
HMP	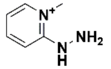	Cortisol, cortisone	TFA in EtOH, 60 °C, 1 h	1000 times increase of detection sensitivity due to a fixed charged quaternary amine	2017 [71]
2-HP	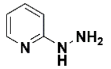	Ketosteroids	In MeOH, 60 °C, 30 min	2-HP is superior not only in chromatographic behavior but also in signal response than the other four reagents	2017 [75]
	**Hydrazides**			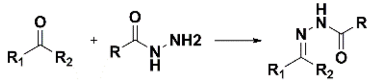	
GT	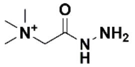	Neurosteroids	FA in MeOH, 37 °C, 30 min	The neurosteroids with highly similar structures derivatized with GT successfully achieved better chromatographic separation	2016 [76]
HTMOB	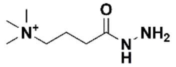	Aldehydes and ketones	FA in MeOH, 20 °C, 30 min	3.3- to 7.0-fold sensitivity enhancement than GT derivatives	2007 [77]
GP	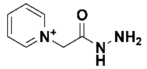	Keto-Steroids	AcOH in MeOH, 60 °C, 10 min	The quaternary ammonium enhanced the efficiency of MS^n fragmentation	2016 [78]
HIQB	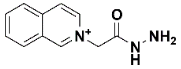	Carbonyl compounds	AcOH in MeOH, 50 °C, 3 h	11-1674 folds sensitivity enhancement	2017 [81]
TMTH	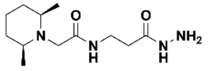	Oxosteroids	AcOH in EtOH/H_2O (7:3), 70 °C, 30 min	Sensitivity enhanced by 14-2755 folds	2014 [83]

Reagents	Structure	Analytes	Derivatization conditions	Comments	Year& Ref.
CHH	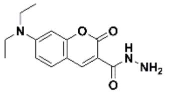	Aliphatic and lipid-bound aldehydes and ketones	In ACN/water (1:1), 37 °C, 1 h	The first method to simultaneously detect aliphatic and lipid-bound aldehydes and ketones	2015 [84]
Glutamic acid-related hydrazide	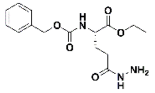	Carbonyl compounds	AcOH in MeOH/H_2O, RT, 120 min	Enhance sensitivity than DNPH owing to its higher ionization efficiency	2018 [85]
TSH	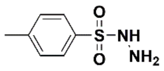	61 aldehydes and ketones	HCl in MeCN, 4°C, 8 min	A suitable substitute for DNPH with similar reactivity and better compatibility with aqueous biological samples	2014 [86]
Dns-Hz	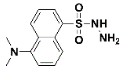	Lipophilic reactive carbonyls	p-TsOH in ACN, RT, 4 h in the absence of light	Enhanced sensitivity but relatively weak isomer separation capability	2015 [87]
	***O*-substituted hydroxylamines**		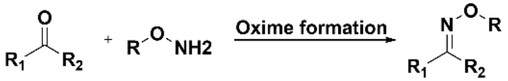		
HA	H_2N-OH	Steroid hormones	In MeOH, 40 °C, 20 min	3.9-202.6 times decrease of LOQs but retention improvement is limited	2019 [88]
		Hexanal and heptanal	During ESI process	Avoid LC separation of *E*-/*Z*- isomers for higher sensitivity	2017 [89]
MOA	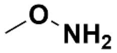	Steroid hormones	In MeOH, 60 °C, 30 min	The excessive MOA can be easily diverted to waste without compromising the ionization efficiency of derivatives	2020 [91]
QAO	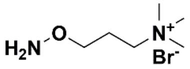	Testosterone and corticosterone	5% AcOH in MeOH, 50 °C, 90 min	10 times higher sensitivity than that of HA	2019 [94]
QDA	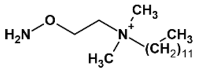	Carbonyl metabolites	2mM DMP in ACN, -80 °C, 3 h	Minimize the degradation of unstable metabolites due to simultaneous quenching and derivatization at -80 °C	2018 [96]
BBHA	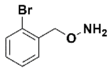	Free carbonyl compounds	In PIPES buffer (0.1M, pH 6.5), 6-8 °C, 1 h	Achieve selective detection based on the presence of the characteristic $^{79}Br/^{81}Br$ isotopic pattern	2015 [97]
Aminooxy-N-(3-perfluorooctyl-propyl) acetamide	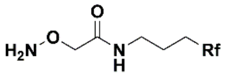	Aldehydes and ketones	In 50% MeOH/H_2O, 70 °C, overnight	Due to the presence of fluorous tag, the derivatives can be extracted from the biological fluids to simplify analysis	2015 [98]

Reagents	Structure	Analytes	Derivatization conditions	Comments	Year& Ref.
Magnetic chemoselective probe	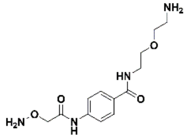	Carbonyl metabolites	In phosphate buffer (50 mM, pH 6.5), 25 °C, 16 h	The chemoselective probe immobilized to magnetic beads can efficiently separate carbonyl metabolites from biological matrix to reduce interference	2020 [100]
	***N*-substituted hydroxylamines**		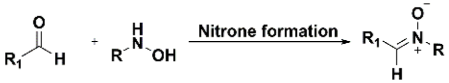		
HAHC	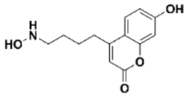	Aldehydes	In acetic acid/ sodium acetate buffer (0.1 M, pH 3.5), 70 °C, 1 h	The first to design and synthesize a novel *N*-substituted hydroxylamine, which overcomes the shortcomings of forming *E*-/*Z*- isomers	2017 [101]
HAMC	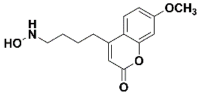	Aldehydes	In acetic acid/ sodium acetate buffer (0.1 M, pH 3.5), 25 °C, 30 min	Mild and efficient labeling in less than 30 min at room temperature	2018 [102]
	**Minoamines**		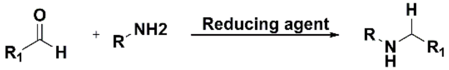		
Aniline	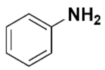	Hexoses	10 % AcOH in MeOH/water (1:1), 40 °C, 30 min; then picoline-borane was added, 30 °C, 45 min	Amine is a weak base that can still have enough unprotonated form to react with sugar at low pH	2017 [105]
AEC	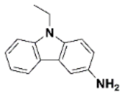	Sugar phosphates	$NaBH_3CN$ and acetic acid in MeOH/water (3:1), 60°C, within 10 min	Shorten derivatization time due to the solid-phase derivatization	2016 [108]
4-APC	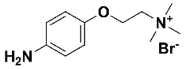	Aldehydes	$NaBH_3CN$ in NH_4Ac buffer (50 mM, pH 5.7), 15 °C, 3 h	The pKa of the aniline moiety is low enough to retain nucleophilic property to ensure the fast reduction of the resulting imine by $NaBH_3CN$	2015 [110]
4-APEBA	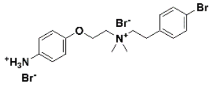	Aldehydes	$NaBH_3CN$ in NH_4Ac buffer (150 mM, pH 5.7), 10 °C, 3 h	Enhance the recognition of metabolites through the characteristic $^{79}Br/^{81}Br$ isotopic pattern	2010 [111]
MPIA	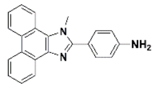	Aliphatic aldehydes	$NaBH_3CN$ in NH_4Ac buffer (pH 5.7), RT, 3 h	Better sensitivity than DNPH, which is probably attributed to the strong proton affinity of the lone-pairs on imidazole nitrogen-atom	2014 [112]
	**Diamines**		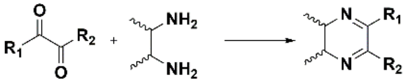		

Reagents	Structure	Analytes	Derivatization conditions	Comments	Year& Ref.
OPD	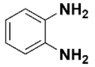	Methylglyoxal	In HCl-DETAPAC, RT, 4h in the dark	Enhanced sensitivity but may have side effects with high level of sugar	2019 [117]
TRI	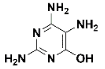	α-Dicarbonyl compounds	In 0.05 M HCl, 85°C, 1 h	Form much less amount of interference compared to OPD	2015 [118]
DABP	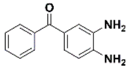	MDA	In phosphate buffer (50 mM, pH 4), 50°C, 30 min	Enhance sensitivities by 50-100 times than DNPH	2017 [119]
	**Others**				
Cysteine	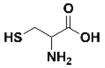	Aldehydes	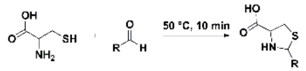	Avoid formation of *E*-/*Z*- isomers	2011 [120]
Acetylacetone	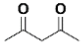	Formaldehyde	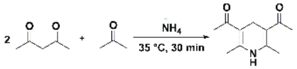	Mild reaction conditions but it is more toxic and not easy to store	2017 [121]
CHD	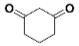	Aldehydes	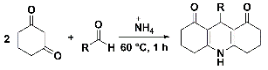	Less toxic and irritant than acetylacetone	2019 [123]
Ammonium sulfite	$(NH_4)_2SO_3$	Aldehydes	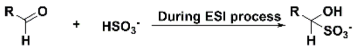	Ammonium sulfite derivatives suffer less matrix effects than LC-PCD-MS method that employs HA	2020 [125]
PQ	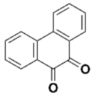	Aliphatic aldehydes	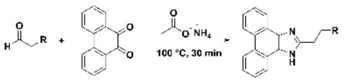	High selectivity for aliphatic aldehydes and 19-1000 times higher sensitivity than MPIA	2016 [126]
Pyridine	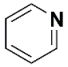	Fatty aldehydes	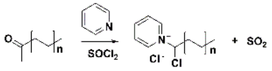	Excess derivatization reagents are less competitive in ionization	2016 [127]
PMP	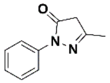	Sugars	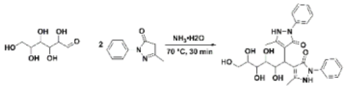	Achieve baseline separation of some sugar isomers within 10 minutes, but can't achieve well separation of xylose and arabinose	2018 [129]
